# Key nutritional factors and interactions during larval development of pikeperch (*Sander lucioperca*)

**DOI:** 10.1038/s41598-019-43491-1

**Published:** 2019-05-08

**Authors:** Najlae El Kertaoui, Ivar Lund, Hospice Assogba, David Domínguez, Maria S. Izquierdo, Sébastien Baekelandt, Valérie Cornet, Syaghalirwa N. M. Mandiki, Daniel Montero, Patrick Kestemont

**Affiliations:** 1 Research Unit in Environmental and Evolutionary Biology (URBE), Institute of Life, Earth & Environment (ILEE), University of Namur, Rue de Bruxelles, 61-5000 Namur, Belgium; 2 Technical University of Denmark, DTU Aqua, Section for Aquaculture, The North Sea Research Centre, P.O. Box 101, DK-9850 Hirtshals, Denmark; 3 Instituto ECOAQUA, Universidad de Las Palmas de Gran Canaria. Grupo de Investigación en Acuicultura (GIA), Muelle de Taliarte s/n, 35200 Telde, Las Palmas, Canary Islands Spain

**Keywords:** Physiology, Biological models

## Abstract

The effects of 8 nutritional variables (Ca/P, Eicosapentaenoic acid (20:5n-3) + Docosahexaenoic acid (22:6n − 3) (EPA + DHA), Arachidonic acid (20:4n − 6) (ARA), Se, vitamins E, C, D and A) were investigated to identify their respective importance and interactions in pikeperch larval development. In this respect, two modalities (low and high levels) of each variable were tested through a fractional factorial experimental design allowing a reduction from 256 (2^8^) to 16 (2^8 – 4^) experimental units. Survival was significantly higher in larvae fed a high Ca/P diet while larval growth was significantly lower in larvae fed the same diet variant, associated with a higher incidence of kyphosis and pectoral anomalies in these larvae. Lordosis and scoliosis seemed to be mostly affected by dietary long chain polyunsaturated fatty acids (LC-PUFAs). A significant interaction was shown between n-3 LC-PUFA and vitamin C on jaw anomalies, while myocyte-specific enhancer factor 2C (*mef2c*) gene expression correlated positively with dietary vitamin C increment. Results also demonstrated an effect of the different nutrients and their interactions on the activity levels of digestive enzymatic activities. The results of the present study highlight the importance of the interactions between Ca/P, LC-PUFAs and vitamins C and E, suggesting their essential roles as key nutritional factors influencing pikeperch larval development.

## Introduction

Pikeperch (*Sander lucioperca*) has been identified as a candidate for diversification with a great potential in European aquaculture industry^1–5^. However, as for some other emerging species, weaning to dry diets remains a major bottleneck for the successful larval rearing, mainly due to the difficulties to valorize efficiently the compound diets, in terms of survival, growth performance and reduced skeletal anomalies. Indeed, the digestive system of early fish larvae is extremely immature, what may correlate with low enzymatic capacities^6–9^. In pikeperch, it has been shown that the structure and functional ability of the digestive tract are affected by the larval developmental stage as well as by the diet composition^10^. In this respect, literature suggested a combined effect of weaning age/size and diets on skeletal anomalies and larval performance in pikeperch^10–12^. Tailored commercial starter feeds do not exist for this species, and feeds used in hatcheries are likely developed for marine species, such as Gemma Micro (Skretting, Norway) and Otohime (Reed Mariculture, California). Hence, during this critical life stage, adequate feeds are needed to fulfil the nutritional requirements of fish larvae.

Literature is scarce on the nutritional requirements of pikeperch; however, in the last decade, some studies have focused on the effects of long-chain polyunsaturated fatty acids (LC-PUFAs) and phospholipids (PLs) on early development of pikeperch larvae^13–18^. Recently, Lund and El Kertaoui *et al*.^19^ highlighted the beneficial effects of a combined supplementation of PL with n-3 LC-PUFA in the form of triglycerides on growth rate, reduced incidence of skeletal anomalies and increased digestive enzymatic capacity. Indeed, improvement in growth rate and digestive capacities have been observed in pikeperch larvae fed high PL levels^10,14^, while a deficiency in n-3 LC-PUFA, especially docosahexaenoic acid (DHA: 22:6n-3), resulted in a higher locomotor activity and alteration in swimming behavior^18^; a higher mortality rate and a decreased salinity tolerance^17,20^. Authors suggested high requirements of pikeperch larvae similar to those of marine carnivorous fish larvae for both phospholipids and LC-PUFAs^10,18^.

Studies in marine fish larvae ranged the optimum levels of n-3 LC-PUFA from 0.05% to 4% on a dry matter basis, in live food or formulated diets^21^. This wide range is most likely due to species-specific requirements. However, literature data also reported different optimal LC-PUFA levels for arachidonic acid (ARA, 20:4n-6); eicosapentaenoic acid (EPA, 20:5n-3) and DHA for the same species and during the same life stage, most likely as a consequence of differences in experimental conditions, especially dietary composition, considering an additional effect of interactions with and between different nutrients. Indeed, the interactions between LC-PUFAs and vitamins, specially vitamins E and/or C, have been reported in several freshwater species, such as walleye (*Sander vitreus*)^22^, beluga (*Huso huso*)^23^, Persian sturgeon (*Acipenser persicus*)^24–26^ and marine fish larvae such as gilthead sea bream (*Sparus aurata*)^27–29^, European sea bass (*Dicentrarchus labrax*)^30^, yellowfin seabream (*Acanthopagrus latus*)^31^ and meagre (*Argyrosomus regius*)^32^. In tandem to the dietary requirement of vitamins (E and C) in fish larvae, they also constitute a part of larval defense against lipid oxidation^33^. Eventually, being an efficient hydrogen donor, vitamin E – for which α-tocopherol has the highest biological activity- reacts as a chain breaking antioxidant^34^ able to compete for peroxyl radicals much faster than any PUFAs. Vitamin C acts as an antioxidant able to regenerate α-tocopherol from tocopheroxyl radical^35–37^. In addition, vitamin C is a cofactor in many biological processes including collagen synthesis^38,39^. Hence, skeletal malformations are common in vitamin C deficient fish^40^. Vitamin A whose antioxidant function has been also described^41^ is among the nutritional causative factors of skeletal anomalies in reared fish together with vitamin D^42^.

During the last years, more considerations have been given to mineral nutrition in fish larvae^43–48^. Among the minerals studied, selenium (Se) has been investigated with respect to its antioxidant role in fish larvae^43,45,49^, likewise, interactions between Se and vitamin E have previously been demonstrated^50,51^. The interaction between calcium (Ca) and phosphorus (P) remains among the most important between minerals. In fact, authors have suggested that dietary Ca/P ratio should be considered as well as individual dietary levels of minerals^52^, since the ratio between Ca and P affects the uptake of calcium^49^. As Ca and P exist in a constant ratio in fish bone^53^, this suggests needs to be maintained in fish feeds^54^.

In general, interactions among nutritional factors can yield antagonistic, additive or synergistic effects. Hence, to increase knowledge of the larval nutritional requirements, macro and micronutrients have to be considered together, such as described in case of interactions between pro and antioxidant nutrients. The fractional factorial design is a practical approach for studying the combined effects of the interactions between various nutrients. Therefore, the aim of the present study was to investigate simultaneously the dietary effect of LC-PUFAs, vitamins and minerals and their interactions using pikeperch larvae as a biological model.

## Results

### Evaluation of the combinations, based on the global score of interest

The highest final weight and SGR were recorded in larvae fed diet 11 (Table 1). Global score calculated by considering the results obtained from husbandry and skeletal anomalies results showed that the combination 3 (diet 3) resulted in the best larval performances (Table 1). High global scores of interest (>3) were also obtained for diets 5, 7, 11. All these experimental treatments contained the lowest dietary Ca/P ratio (0.6); in addition, these treatments (3, 7, 11) with the exception of diet 5 are characterized by the highest n-3 LC-PUFA content (EPA + DHA = 3.5%). The lowest weight and SGR were recorded in larvae fed diet 10 which combined the highest dietary Ca/P ratio and the lowest EPA + DHA, followed by treatments 8, 12, and 6 which were characterized by the highest dietary Ca/P ratio (Table 1). In addition, low global scores of interest were observed in treatments 15, 16, grouping the combination of high EPA + DHA, high vitamin E and high vitamin A.

**Table 1:** Husbandry and anomalies results of the different combinations tested in the experiment.

Diet	Variables studied										Global score of interest	Rank of the global score
	Final weight (mg)	SGR (day^−1^)	Cannibalism (%)	Mortality (%)	Survival (%)	Anomaly abundance (%)						
						Pectoral elements	Kyphosis	Lordosis	Scoliosis	Jaws		
1	82.47	16.67	30.83	40.40	27.48	32.00	2.00	38.00	18.00	30.00	−0.49	9
2	71.40	15.56	35.86	20.32	42.35	28.57	4.08	46.94	28.57	24.49	0.04	7
3	101.02	18.23	46.11	26.35	27.60	20.00	2.00	26.00	4.00	18.00	6.92	1
4	74.26	15.86	32.66	16.87	49.51	45.83	10.42	47.92	25.00	39.58	−1.90	11
5	78.94	16.33	26.56	29.80	42.36	12.24	6.12	42.86	18.37	4.08	6.17	2
6	68.10	15.19	37.34	15.61	45.60	48.00	2.00	26.00	16.00	42.00	−0.62	10
7	90.72	17.40	53.28	20.62	25.66	20.41	0.00	44.90	12.24	18.37	3.00	4
8	64.19	14.74	26.52	24.85	47.11	22.00	14.00	46.00	20.00	22.00	0.31	6
9	86.35	17.02	42.19	29.43	27.96	41.51	0.00	54.72	16.98	37.74	−2.51	13
10	61.46	14.40	30.66	30.84	36.65	35.29	5.88	52.94	27.45	27.45	−4.58	14
11	103.52	18.42	32.47	31.09	35.58	35.29	1.96	25.49	9.80	35.29	5.30	3
12	67.92	15.17	40.53	22.89	35.74	28.00	14.00	50.00	16.00	24.00	−2.47	12
13	74.06	15.84	27.26	38.15	33.09	34.00	4.00	34.00	12.00	32.00	−0.37	8
14	82.18	16.64	25.33	43.59	28.65	34.00	6.00	24.00	10.00	28.00	1.08	5
15	71.38	15.56	14.14	50.31	33.75	34.62	15.38	59.62	25.00	26.92	−4.92	15
16	88.94	17.25	47.62	15.92	36.57	24.49	24.49	73.47	46.94	24.49	−4.94	16

### Evaluation of the global effect of combination by PCA

**Figure 1:**
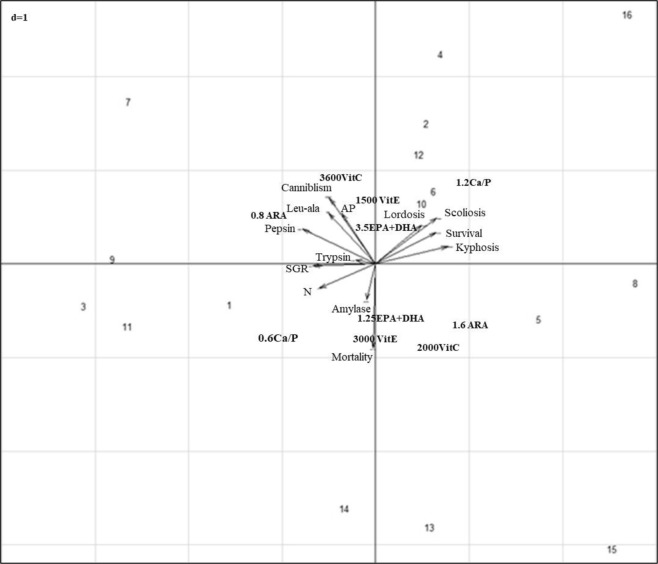
Projections of outputs and combinations (1–16) on the two first axis of the principal component analysis. The plans 1 and 2 of the PCA explain 50.8% of the projected inertia. Axis 1 (inertia 29.2%), axis 2 (inertia 21.6%).

The results of the principal component analyses indicate that the husbandry and anomaly responses are related to the dietary content (Fig. 1). The plans 1–2 of the PCA explain 50.8% of the inertia (total variance). Indeed, the axis 1 (Fig. 1), characterized by the vectors scoliosis, lordosis, survival and kyphosis, and the modality 1.2 Ca/P, was especially represented by the dietary treatments 4, 8, 16 in which both kyphosis and survival rate were higher than the global average rate recorded (Table 1). On the opposite, on this axis, the treatments 3 and 11, in which a high final weight and SGR were recorded (Table 1) were characterized by the vectors final weight, SGR, aminopeptidase specific activity (N), and the modality 0.6 Ca/P. In addition, the increase in dietary vitamin E content up to 3000 mg kg^−1^ together with the use of low EPA + DHA (1.25%) dietary content significantly affected mortality rate and amylase activity.

### Results presented by factor effect

#### Husbandry and skeletal anomalies

**Figure 2:**
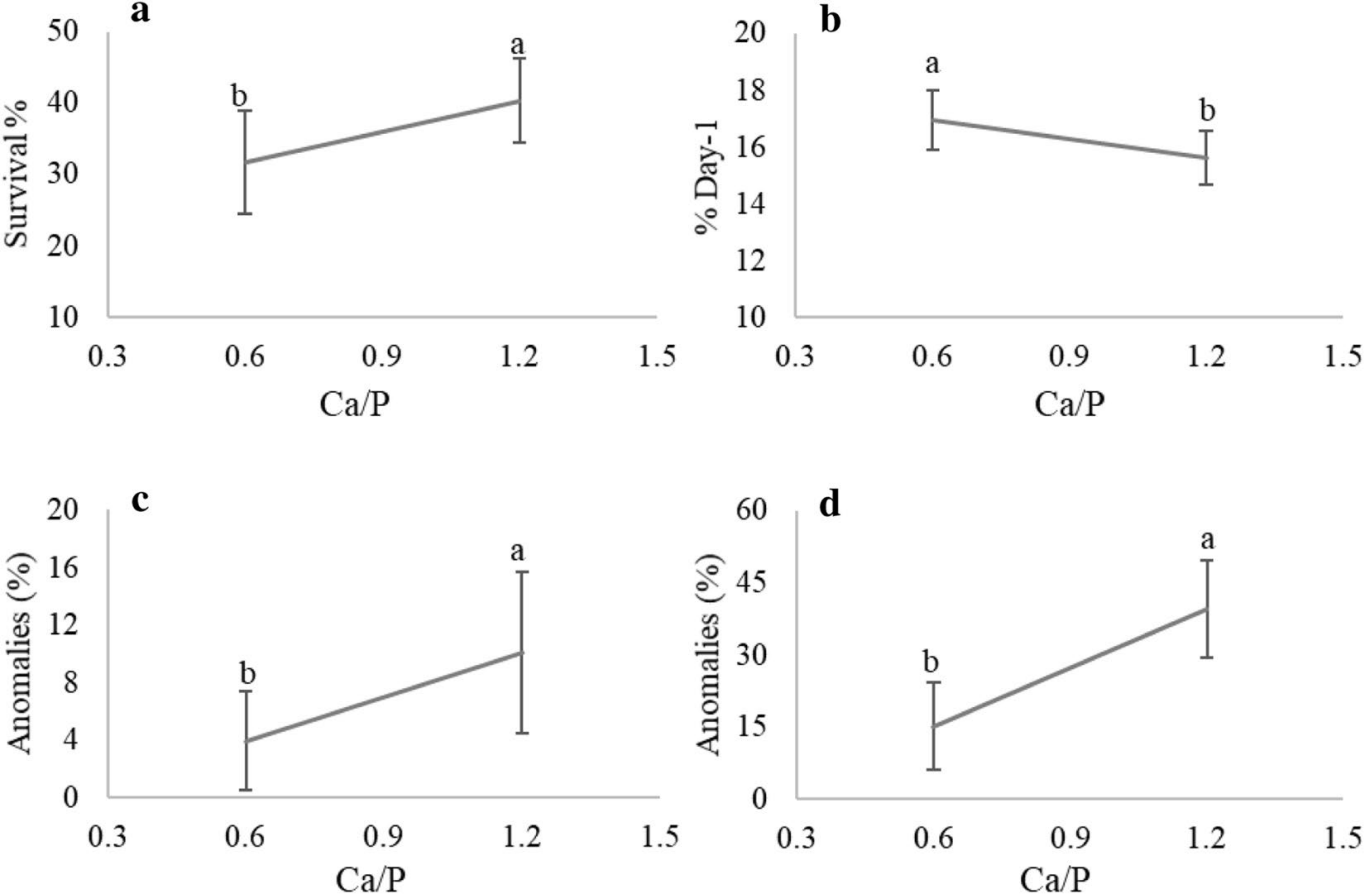
Effect of dietary Ca/P on husbandry variables and anomalies: (**a**) larval survival rate (%); (**b**) specific growth rate (SGR, %day-1); (**c**) kyphosis rate (%); (**d**) pectoral element anomalies rate (%). Only graphs with significant effects are shown. Results are expressed as the mean ± SD (n = 8). Different letters denote statistically significant differences between treatments.

The detection of the potentially active effects of the tested factors on the husbandry, and skeletal anomalies variables as given by Daniel graphics and followed by ANOVA showed significant effects of Ca/P ratio on several endpoints. Survival rate was higher in larvae fed high Ca/P (p = 0.017) (Fig. 2a). Contrary, final weight and specific growth rate (SGR) decreased significantly in larvae fed high Ca/P (p = 0.021 and 0.018 respectively) (Fig. 2b). In addition, the prevalence of kyphosis and pectoral element anomalies increased by the dietary Ca/P elevation (p = 0.0006) (Fig. 2c,d).

**Figure 3:**
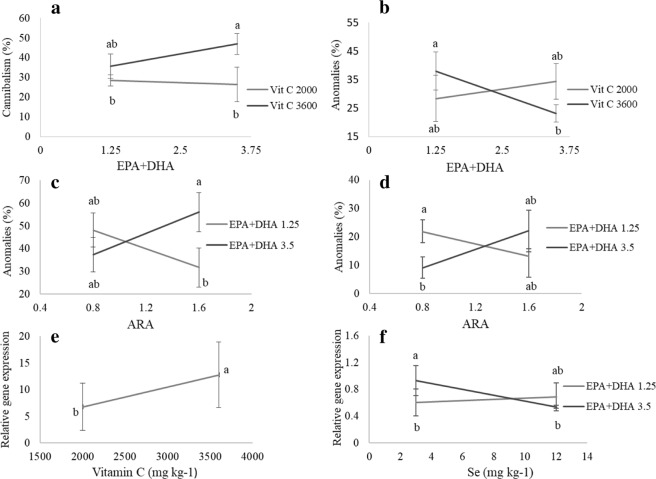
Effect of dietary nutrient factors on cannibalism, larval anomalies and related gene expression: (**a**) EPA + DHA and vitamin C interaction effect on cannibalism rate; (**b**) EPA + DHA and vitamin C interaction effect on jaw anomalies; (**c**) EPA + DHA and ARA interaction effect on lordosis; (**d**) EPA + DHA and ARA interaction effect on pre-hemal scoliosis; (**e**) *mef2c2* and (**f**) *twist2* expression measured in pikeperch larvae. Only graphs with significant effects are shown. Results are expressed as the mean ± SD (single effect: n = 8; interaction effect: n = 4). Different letters denote statistically significant differences between treatments.

Combined effects of n-3 LC-PUFA (EPA + DHA) and vitamin C had a clear direct incidence in jaw anomalies and cannibalism. The dietary increase in EPA + DHA reduced jaw anomalies in larvae fed 3600 mg vitamin C (p = 0.033) (Fig. 3b), while the same combination resulted in higher cannibalism rate (p = 0.05) (Fig. 3a).

Results of lordosis and pre-haemal scoliosis presented a significant interaction between EPA + DHA and ARA dietary contents (p = 0.0081 and 0.0071 respectively). The increase of EPA + DHA (3.5%) seemed to reduce the prevalence of scoliosis in larvae fed 0.8% ARA (Fig. 3d), while there was no significant effect at high level of ARA (1.6%). Besides scoliosis, high levels of EPA + DHA and ARA increased lordosis (Fig. 3c), while the decrease in EPA + DHA with the high ARA level reduced skeletal anomalies (Fig. 3c,d). No statistically confirmed effects of the dietary nutrient factors on the normal specimen rate (larvae without severe anomalies) were detected, this latter ranging between 11.76% (larvae fed diet 10) and 56% (larvae fed diet 3).

Myocyte enhancer factor-2 (*mef2c2*) was overexpressed in larvae fed high vitamin C levels (Fig. 3e). In addition, *twist* expression was highest when pikeperch larvae were fed the combination low Se and high n-3 LC-PUFA; but dietary Se supplement resulted in a decrease of *twist2* expression in larvae fed high n-3 LC-PUFA (Fig. 3f).

#### Activity of digestive enzymes

**Figure 4:**
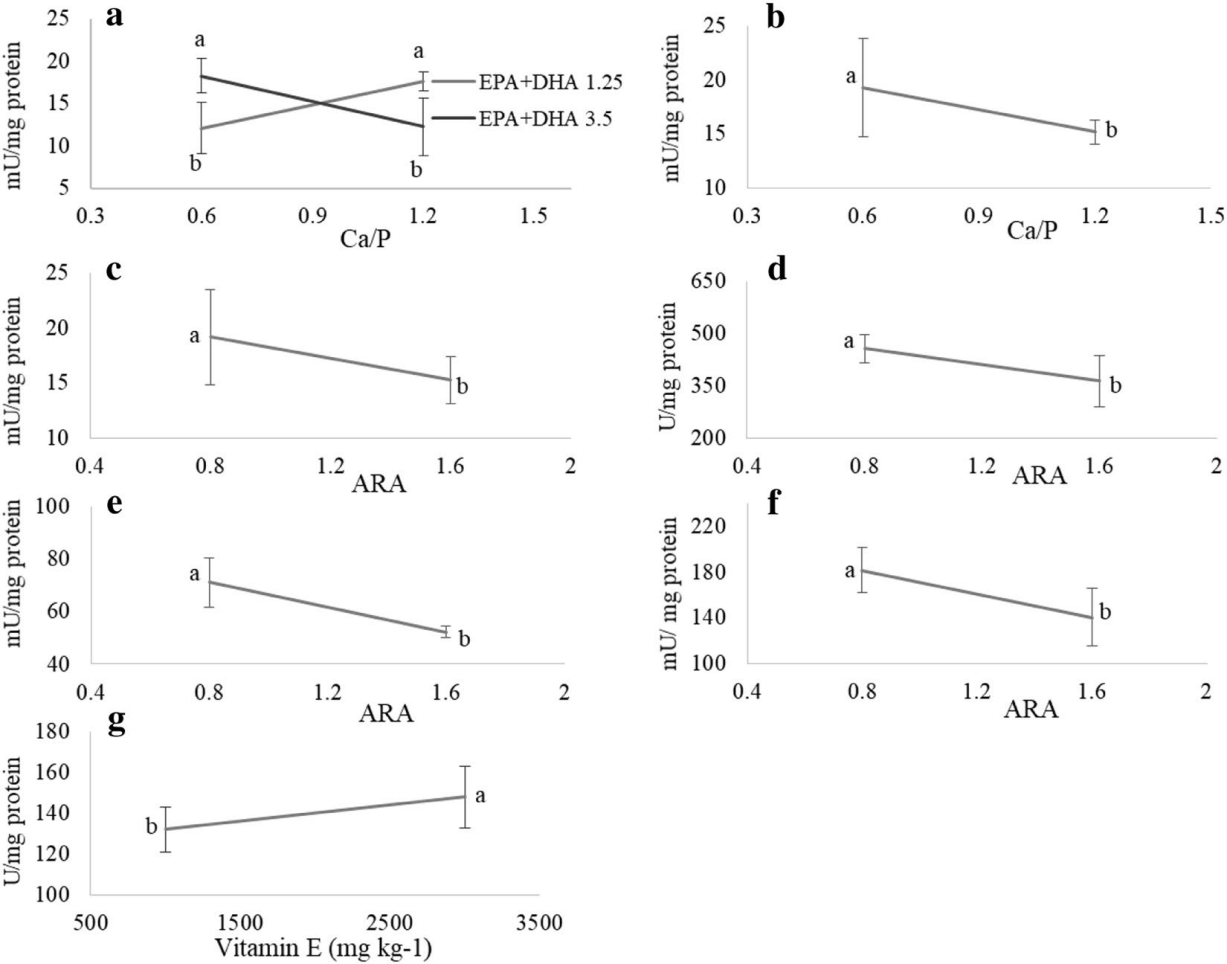
Digestive enzymatic activity (**a**) trypsin; (**b**,**c**) aminopeptidase; (**d**) leucine alanine; (**e**) alkaline phosphatase; (**f**) pepsin; (**g**) amylase) of 39 dph pikeperch larvae fed the different experimental diets. Only graphs with significant effects are shown. Results are expressed as the mean ± SD (single effect: n = 8; interaction effect: n = 4). Different letters denote statistically significant differences between treatments.

A differential pattern in the ontogenetic development of digestive enzymes was observed dependent on the dietary content. High dietary n-3 LC-PUFA content enhanced trypsin activity in larvae fed low Ca/P level (Fig. 4a). On the contrary, this elevation of dietary n-3 LC-PUFA resulted in a decrease in trypsin activity in larvae fed 1.6 Ca/P level (p = 0.0044) (Fig. 4a). A higher aminopeptidase activity (N) was also observed in larvae fed low Ca/P (p = 0.026) (Fig. 4b). Aminopeptidase activity was also significantly lower in larvae fed high ARA level (p = 0.038) (Fig. 4c). Similarly, leucine alanine peptidase (leu-ala), alkaline phosphatase (AP) and pepsin specific activities were negatively correlated with ARA levels (p = 0.023; 0.0017 and 0.0053; respectively) (Fig. 4d–f), while specific activity of amylase increased with dietary vitamin E elevation (p = 0.012) (Fig. 4g).

#### Larval content in fatty acids

**Figure 5:**
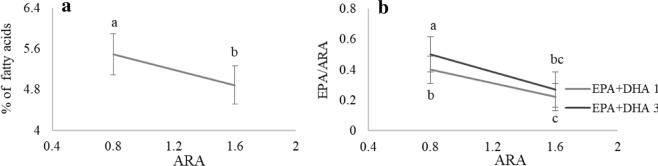
Larval fatty acid content of 39 dph pikeperch. (**a**) EPA larval content and (**b**) EPA/ARA ratio in larvae fed different dietary ARA. Only graphs with significant effects are shown. Results are expressed as the mean ± SD (single effect: n = 8; interaction effect: n = 4). Different letters denote statistically significant differences between treatments.

In terms of fish fatty acid content, DHA, EPA and the total n-3 LC-PUFA were significantly higher in the group of larvae fed the high n-3 LC-PUFA treatments (3.5%) (Supplementary Table S6). On the other hand, the increase in dietary ARA seemed to reduce EPA content in larval tissues (Fig. 5a) as well as EPA/ARA ratio (Fig. 5b). No statistically confirmed effects of vitamins or minerals on larval fatty acids content were detected.

#### Histological study

**Figure 6:**
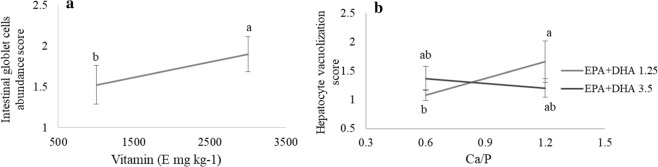
(**a**) Intestinal goblet cells and (**b**) hepatocyte vacuolization observed in pikeperch larvae fed different diets. Score (1) not observed; (2) mild vacuolization or goblet cells presence; (3) severe vacuolization or goblet cells presence. Only graphs with significant effects are shown. Results are expressed as the mean ± SD (single effect: n = 8; interaction effect: n = 4). Different letters denote statistically significant differences between treatments.

Histological changes were observed in the anterior intestine samples from 3000 mg kg^−1^ vitamin E diets group, that were characterized by an increased density of goblet cells (Fig. 6a) (p = 0.0044). Regarding the liver, high Ca/P resulted in an increase of lipid vacuole accumulation in larvae fed low n-3 LC-PUFA level (Fig. 6b) (p = 0.0035). Larvae fed diets combining low n-3 LC-PUFA and low Ca/P contents displayed very condensed hepatocytes with centered nucleus and marked cytoplasm staining, with a scarce deposition of lipid reserves (Supplementary Fig. S1). On the contrary, the increase in Ca/P did not affect the lipid vacuole deposition in larvae fed high LC-PUFA levels (Fig. 6b). No other histopathologies were observed in larval tissue.

## Discussion

Although the fractional factorial design is considered as a practical approach permitting to identify the most influencing factors as well as the evaluation of the possible interactions between the tested factors^55–61^, such design regrouping more than 3 nutrients concurrently are rarely used in fish^55,62^. To our knowledge, the present study represents a first attempt to evaluate the simultaneous effects of selected fatty acids, vitamins and minerals using a multifactorial approach in fish larvae.

Our finding shows that specific endpoints describing larval performance are specifically influenced by different nutrients and doses. The evaluation of the combinations, based on the global score of interest, suggested a profound effect of Ca/P ratio, n-3 LC-PUFAs and vitamin E. In fact, the four best dietary combinations are commonly characterized by a low dietary Ca/P ratio (0.6) and a high EPA + DHA content (3.5%), while the elevation of dietary vitamins E and A (3000 mg kg^−1^ and 30000 IU respectively) and LC-PUFAs (EPA + DHA and ARA) resulted in a lower global score of interest. In the present study, no potential main effect was assigned to vitamins D and A. Similarly, except its related effect to n-3 LC-PUFA on *twist* expression, no significant differences were recorded among larvae fed different Se levels. Minor effects of micronutrients on gross fish performance have also been suggested in Atlantic salmon (*Salmo salar, L*.) by Hamre *et al*.^55^.

Our results vary according to the endpoint considered. Indeed, Ca/P ratio appears as a determining nutrient for pikeperch larvae considering the significant decline in growth as well as the increase in the incidence of skeletal anomalies (especially kyphosis and pectoral element anomalies) recorded in larvae fed high Ca/P. Kestemont *et al*.^11^ already suggested the need for a low Ca/P ratio in pikeperch larval diets, below those usually found in commercial feeds formulated for marine fish larvae. In this study, growth was probably reduced due to the higher incidence of bone anomalies, since skeletal deformation may affect various physiological and behavioral performances of fish larvae such as swimming, feed intake and feeding efficiency^63^. Taking into account the total phosphorus level, which was about two times higher in 0.6 Ca/P than in 1.2 Ca/P feeds, we can speculate that the increase in skeletal anomalies induced by the dietary Ca/P elevation indicates that the diets with high Ca/P ratio were not able to fulfil the phosphorus requirements of pikeperch larvae. In accordance with our findings, high incidence of kyphosis is often reported as a typical consequence of phosphorus deficiency in fish larvae^64^. Based on a rather large review of the literature (including 25 studies on different finfish species), Antony Jesu Prabhu *et al*.^65^ reported that the estimated minimal dietary available P content is 3.5 g kg^−1^ DM. In our study dietary Ca/P ratio induced opposite effects on survival and on growth. The high survival rate in larvae fed the high Ca/P ratio could be associated to the increment of the efficiency of P utilization in low dietary available P feeds^66^. While the low survival rate governed also by the cannibalistic behavior of pikeperch larvae could be responsible for the higher growth rate recorded in low Ca/P groups.

PCA analysis also associated the increase in mortality with the high vitamin E intake (3000 mg kg^−1^). High vitamin E levels have been suggested to act as pro-oxidant^67,68^, which explains the lowest score of interest recorded in larvae fed diet 16 combining high Ca/P and high vitamin E levels, taken into consideration the increased antioxidant defense ability by optimal dietary phosphorus^69^. Thus, contrary to what is observed in marine fish larvae in which a beneficial effect of dietary vitamin E elevation – up to 3000 mg kg^−1^- has been recorded^27,29,30,32^^,^ the vitamin E levels chosen in this study seem to be higher than the need of this species. Similarly, an increase in mortality and tissue lipid oxidation has been reported in Atlantic salmon and Atlantic halibut (*Hipoglossus hipoglossus*) juveniles fed high levels of vitamin E in the absence of a sufficient amount of vitamin C^33,67^. High dietary vitamin E levels resulted in low growth and death in rainbow trout (*Oncorhynchus mykiss*)^70^, equally a negative effect of high dietary vitamin E levels on growth was also observed in parrot fish (*Oplegnathus fasciatus*)^71^. Furthermore, the toxic effects have been reported in several species such as brook trout fry (*Salvelinus fontinalis*)^72^, African catfish (*Clarias gariepinus*)^73^, and rainbow trout^74^.

In the present study, high vitamin C dietary content seemed to be efficient in reducing the incidence of jaw anomalies when high levels of n-3 LC-PUFA were included in the diets, pointing out the antioxidant function of this vitamin^30,75^. In this sense, Izquierdo *et al*.^28^ highlighted the relationship between the appearance of anomalies in skeletal elements developed from a cartilaginous precursor and the increased oxidative status in seabream larvae fed high DHA levels. A possible explanation may be related to the dietary effect of ascorbic acid on the ossification of cartilaginous- origin bone process, since ascorbic acid affects collagen synthesis in structural organs such as cartilage and bone^76^. Hence, impaired biosynthesis of collagen resulted in skeletal malformations in ascorbic acid deficient fish^40^. Meanwhile, high vitamin C associated with high EPA + DHA level resulted in higher cannibalism rate, probably because of the decrease in the incidence of jaws anomalies. Kestemont *et al*.^11^ also linked the incidence of jaw anomalies to the weaning age and the dietary intake, especially of vitamin C, being deficient in the non-enriched *Artemia* nauplii. Interestingly, in the present study, an increase in dietary vitamin C resulted in the overexpression of *mef2c2* (myocyte enhancer factor-2) expression, providing an additional support to the role of vitamin C intake in the ossification process and collagen synthesis in fish larvae. The role of *mef2c* in promoting precocious chondrocyte hypertrophy and ossification of endochondral bones has been reported by Arnold *et al*.^77^ and Potthoff and Olson^78^. Actually, *mef2c* acts as an essential, early regulator of bone development by orchestrating transcriptional and cell-cell signaling events involved in chondrocyte hypertrophy which is necessary for bone vascularization, osteoblast differentiation and endochondral ossification. Selenium has also been considered to be related closely to endochondral ossification^79^. *Twist2* expression suggested that n-3 LC-PUFA and Se act synergistically, likewise, *twist2* down expression recorded in larvae fed high n-3 LC-PUFA with dietary Se supplement may reflect the antioxidant role of Se in pikeperch larvae, since the twist2 gene antagonizes osteoblast formation^80,81^. Twist2 is a runx2 inhibitor and suppresses runx2 by interacting with its DNA binding domain, and the osteoblast differentiation occurs only after *twist2* gene expression decreases^82,83^. Hence, authors suggested that the expression of *twist* during early fish ontogeny could be used as an indicator of skeleton malformation^84,85^. Meanwhile, in the present study, lordosis and pre-haemal scoliosis were mainly affected by dietary LC-PUFAs. Feeding high dietary LC-PUFAs (diets combining high ARA and EPA + DHA levels) led to an increase in both anomalies (Fig. 3c,d). Excessive amounts of PUFA have been suggested to accelerate osteoblasts differentiation, causing supranumerary vertebrae in sea bass larvae^86^. In our study, the richness in LC-PUFAs could result in a high level of lipid peroxidation. Furthermore, the increase in dietary ARA had a differential effect on skeletal anomalies depending on the EPA + DHA levels. In this regard, a possible explanation could be related to EPA + DHA/ARA ratio, suggesting the importance of a balanced n-3 LC-PUFA/n-6 LC-PUFA ratio in this species. The importance of a balanced level of n-3 and n-6 PUFA for a proper skeletogenesis has been proven in other species^21,87^. In flatfish, authors linked the dietary imbalances in EPA and ARA to the disruption of bone formation and osteoblast differentiation in skeletal tissues, bone remodeling problems in the cranial region and reduction of bone mineralization^88^. This sensitivity to dietary EFA ratios, in particular the balanced proportion among EPA and ARA acids, is related to the fact that both acids are precursors for highly bioactive eicosanoids – especially PGE2 and PGE3 – for which the effects on bone metabolism and osteoblasts regulation have been well reported^88–92^. Thus, although PGE levels were not measured in this study, it can be hypothesized that the anomalies observed indicate a disturbance of bone development, probably due to the prostaglandin imbalance especially the PGE2/PGE3 ratio, such as suggested in previous studies^88–93^. This is in agreement with the fact that, in the present study, the increase in dietary ARA seemed to reduce EPA content in larval tissues; denoting a selective deposition and retention of LC-PUFAs in pikeperch larvae, likely due to the inhibition of EPA incorporation by dietary ARA^94^, which may also result in an alteration of prostaglandin production as suggested above in favor of the more biologically active PGE3 involved in pro inflammatory properties. Actually, the relation among dietary EPA and ARA has been proposed to be a critical factor for larval performance due to competitive interaction among them^89,95,96^.

Previous studies showed that n-3 LC-PUFAs are potent stimulators of cholecystokinine (CCK) secretion^97^. In our study, effects of EPA + DHA levels on trypsin activity may reflect the endocrine modulation of the pancreatic digestive function, which is regulated by CCK^98–100^. In fact, trypsin is secreted as a trypsinogen activated by an enterokinase requiring Ca ions^101^. Consistently, the better utilization of Ca under specific condition of LC-PUFA and Ca/P should be further investigated. The same interaction was found in hepatocyte vacuolization. Fatty liver has been considered as an expression of the impaired lipid metabolism^102^. High intake of LC-PUFAs (mainly EPA and DHA) prevents lipid accumulation^103^. Hence, we speculate that the decrease in dietary Ca/P may enhance lipid utilization in n-3 LC-PUFA deficient fish larvae. In mice, authors found that the dietary phosphorus restriction (in the form of mono-potassium phosphate KH_2_PO_4_) resulted in hepatic lipid accumulation^104^. Moreover, the opposite interaction found between EPA + DHA and Ca/P- on trypsin specific activity as well as the hepatic fat accumulation- could be linked to the effect of Ca/P on growth. On the other hand, the immature intestine of marine fish larvae, has been suggested as a risk for P deficiency^49^. In this study, high Ca/P dietary level - which was also characterized by the low level of dietary P – led to a decrease in aminopeptidase specific activity. Considering the profound Ca/P effect on growth, we hypothesize an enhancement of gut maturation in larvae fed low Ca/P ratio, as indicated by the increase in brush border enzymatic activities, these latter reflecting the normal maturation process of enterocytes in fish larvae^12,105^. Analysis of factor effects as well as principal component analysis linked the decrease in specific activity of pepsin, leucine alanine and brush border enzymes (alkaline phosphatase and aminopeptidase) to the dietary ARA elevation. In this respect, Yuan *et al*.^106^ suggested that the use of a diet containing a moderate level of dietary ARA promotes the maturation of enterocytes in larval tongue sole. Therefore, the high ARA level used in our study may have delayed the enterocyte maturation, supporting its potential involvement in the regulation of the digestive tract development in pikeperch larvae. In addition, dietary vitamin E elevation resulted in an increase in amylase specific activity. Vitamin E supplementation resulted in significantly higher muscle glycogen levels in mammals^107^. Consistently, Ma *et al*.^108^ attributed the increase in amylase activity to the high level in glycogen in copepods distributed to large yellow croaker (*Pseudosciaena crocea*) larvae, since amylase is stimulated by glycolytic chains, glycogen, and starch in fish larvae^109^. In addition, dietary vitamin E elevation resulted in an increased incidence of goblet cells secreting mucins. These results suggest a higher mucus production in fish fed high vitamin E level, that would contribute to explain the observed increase in amylase specific activity. Increased density of goblet cells has been also observed in fish fed immunestimulants such as mannan oligosaccharides^110,111^, whereas vitamin E is considered as a key immune stimulating factor in fish^112,113^.

From the different endpoints analyzed in this multifactorial fractional design, it appears that the directive factors for larval performance, as far as the husbandry aspects and the incidence of skeletal anomalies are concerned, are the Ca/P ratio and the LC-PUFAs dietary contents. Amongst vitamin nutrients, vitamins E and C appear as the major micronutrients of interest for pikeperch larvae.

## Materials and Methods

### Ethical standards

All procedures and protocols were approved by the local Ethic Committee for Animal Research of the University of Namur, Belgium (Protocol number: 16271 KE) in accordance with national and international regulations for the use of animals in scientific experimentation.

### Experimental design

Eight nutrient variables (Ca/P, EPA + DHA, ARA, Se, vitamins A, C, D and E) were tested at two levels of diet inclusion (Supplementary Table S1). With a full factorial design with 8 factors at 2 levels, 256 (2^8^) treatments would be required for the calculation of the main effects and all the interactions. Therefore, the experiment was carried out as a 2^8 – 4^ reduced factorial design (Table 2). With such fractional factorial design, the number of combinations (treatments) is reduced from 256 (2^8^) to 16 (2^4^). To generate the experimental design, an alias structure was selected (Supplementary Table S2), determining which effects are confounded with others^55–57^. In this way, it is possible to calculate main effects separated from each other and from the effects of two-factor interactions^55,57,58,114^. The main advantage of this approach is that it considers simultaneously the impact of a large number of interrelated nutritional factors using a limited number of experimental units. With a 2(^8 – 4^) reduced factorial design, each of the 16 combinations was tested once but each level of every factor was repeated eight times (Table 2).

**Table 2:** Experimental factors-modalities (Diet = experimental conditions).

Diets	Ca/P	EPA + DHA %	ARA %	Vitamin E mg/kg	Vitamin D IU/kg	Vitamin C mg/kg	Vitamin A IU/kg	Se mg/kg
1	0.6	1.25	0.8	1000	2800	2000	8000	3
2	1.2	1.25	0.8	1000	28000	3600	8000	12
3	0.6	3.5	0.8	1000	2800	3600	30000	12
4	1.2	3.5	0.8	1000	28000	2000	30000	3
5	0.6	1.25	1.6	1000	28000	2000	30000	12
6	1.2	1.25	1.6	1000	2800	3600	30000	3
7	0.6	3.5	1.6	1000	28000	3600	8000	3
8	1.2	3.5	1.6	1000	2800	2000	8000	12
9	0.6	1.25	0.8	3000	28000	3600	30000	3
10	1.2	1.25	0.8	3000	2800	2000	30000	12
11	0.6	3.5	0.8	3000	28000	2000	8000	12
12	1.2	3.5	0.8	3000	2800	3600	8000	3
13	0.6	1.25	1.6	3000	2800	3600	8000	12
14	1.2	1.25	1.6	3000	28000	2000	8000	3
15	0.6	3.5	1.6	3000	2800	2000	30000	3
16	1.2	3.5	1.6	3000	28000	3600	30000	12

### Rearing conditions

A batch of pikeperch larvae 3 dph (day post hatching) was obtained from breeders held in Viskweekcentrum Valkenswaard located in Leende, The Netherlands. The initial larval rearing was carried out in two tanks (500 L rectangular tanks with a water depth of 30 cm) from 3 dph until the weaning period. Larvae were fed *Artemia* nauplii enriched with DHA Protein Selco® (INVE, Dendermond, Belgium) each hour (from 8:00 am to 6:00 pm) until 17-day old. This first rearing phase was followed by a co-feeding period from 18 to 24 dph using *Artemia* nauplii and a mixture of the 16 experimental dry diets (200–400 µm pellets). The multifactorial experiment started on 25 dph with completely weaned larvae in order to avoid any bias due to habituation to dry feed. Sixty larvae were randomly sampled and weighed to estimate the initial body weight (9.44 ± 4.42 mg). The 25 dph larvae were randomly distributed into 16 experimental tanks (rectangular aquarium with a water volume of 90 L) with a density of 770 larvae tank^−1^ and fed one of the experimental diets (mixture of 200–400 µm and 400–700 µm pellets) for 14 days. The system was based on flow through and all tanks were supplied with filtered fresh water at a rate of 8% h^−1^ to ensure water renewal and to maintain a high water quality during the experiment. Water was continuously aerated by using an airstone in each tank. Temperature and oxygen were daily measured; average water temperature was 19.5 ± 0.4 °C and dissolved O_2_ averaged 7.8 ± 0.32 mg. Photoperiod was kept at 12 h light: 12 h dark. Tanks were manually cleaned once daily between 3:00–6:00 pm by siphoning allowing a daily counting of larval mortality.

### Diets and feeding

Sixteen isonitrogenous and isolipidic diets containing different levels of Ca/P, EPA + DHA, ARA, Se, vitamin A, C, D and E were formulated and fabricated by SPAROS S.A. (Portugal) as cold extruded feed pellets of 200–400 µm and 400–700 µm. Experimental diets were formulated (Supplementary Table S3) using a mix of oil as sources of EPA, DHA and ARA to reach the required fatty acid content and to equalize the lipid content in each diet. Rovimix A, Lutavit C, Rovimix D3 and Lutavit E, were used as vitamin sources of vitamins A, C, D and E respectively. Selplex-Se yeast was used as a source of Se, while Ca/P levels were obtained by changing the P levels in diets using NaH_2_PO_4_ as source of P (Supplementary Table S3). The proximate composition of the main important nutrients and vitamins/minerals is shown in Supplementary Table S3. Diets were supplied manually every 45 min from 8:00 am to 6:00 pm. Dry feeds supplied was maintained at 25% of the expected larval biomass in the 1^st^ week and 10–15% during the last week. The experimental diets were tested in the factor-modality design (Table 2) which represented a unique variant nutrient combination.

### Samplings and analyses (larval performance)

Final survival was calculated by individually counting all living larvae at the end of the experiment, and expressed as the percentage of the initial numbers of fish. The apparent mortality was calculated by adding the daily counted dead larvae. Type I cannibalism (dead fish showing signs of cannibalism, i.e. fish partly consumed by a cannibal) was not observed. Thus, only missing larvae due to type II cannibalism (i.e. fish completely ingested, usually head first, by a cannibal) were considered to estimate the mortality rate due to cannibalism. All sampled larvae were sedated with tricaine methanesulfonate (MS-222). Growth was determined by measuring wet body weight of 40 larvae per tank at the end of the experiment. Specific daily growth rate SGR (% day^−1^) was calculated according to the equation: SGR = 100 (Ln (final average body weight of sampled larvae) − Ln (initial average body weight of sampled larvae))/number of feeding days.

### Skeleton anomalies and related gene expression

**Figure 7:**
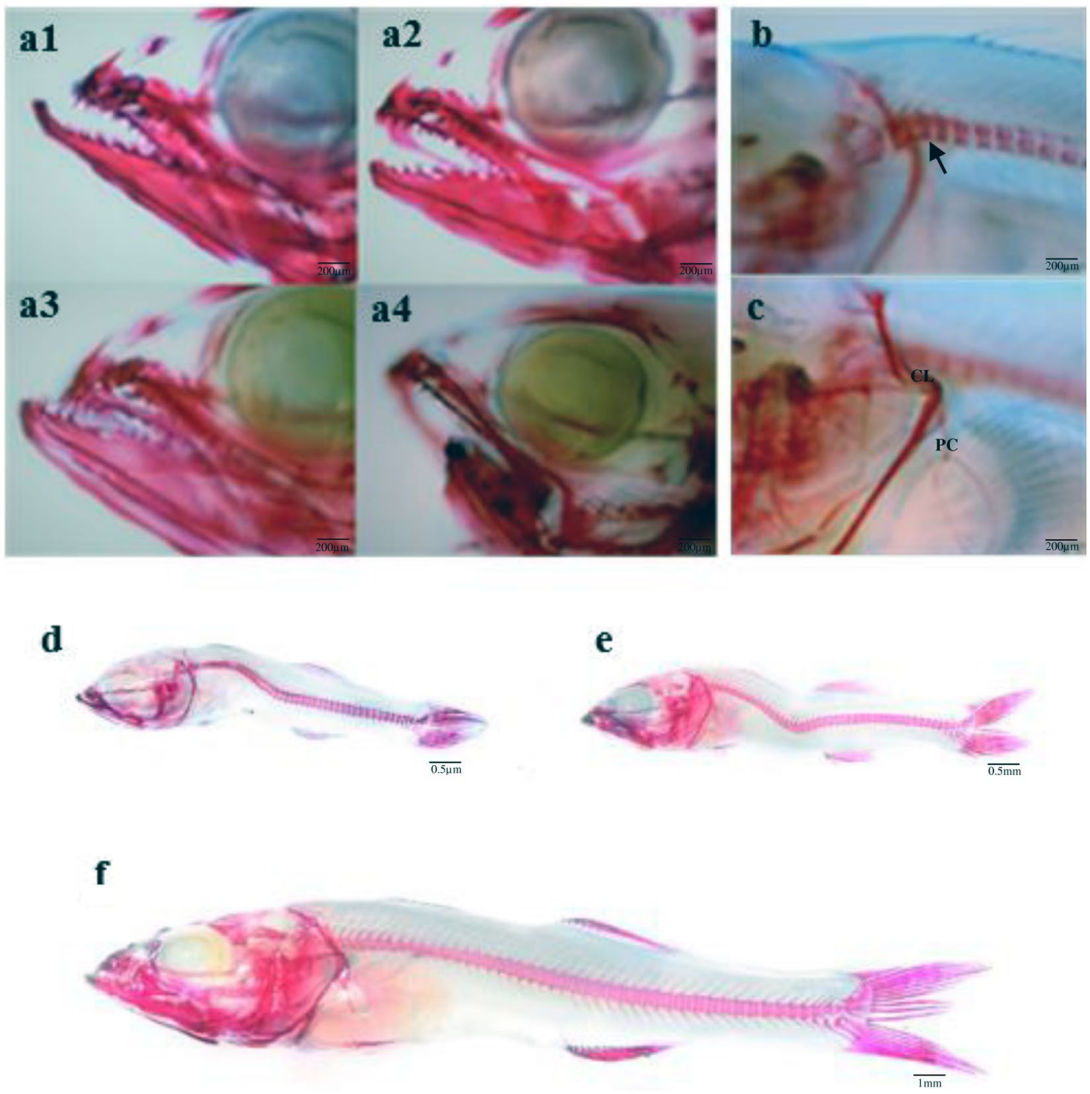
Examples of some skeletal anomalies observed in 39 dph pikeperch *sander lucioperca* larvae. (**a**) Larvae showing different jaw anomalies (a1: lower jaw increment, a2: normal jaws morphology, a3: upper jaw reduction, a4: lower jaw reduction). (**b**) Larvae showing a cephalic kyphosis (arrow). (**c**) Pectoral element anomalies (CL) cleithrum (PC) post-cleithrum. (**d**) larvae showing a severe scoliosis. (**e**) Larvae showing a severe lordosis. (**f**) Normal skeleton of pikeperch larvae.

To determine the presence of skeletal anomalies, 50 larvae per tank were fixed and stored in buffered (10% phosphate) formalin at the end of the experiment. Staining procedures with alizarin red and alcian blue were conducted to evaluate skeletal anomalies following a modified method from previous studies^28^. Classification of skeletal anomalies was conducted according to Boglione *et al*.^115^. Anomalies were expressed as frequency of total severe anomalies and specific anomalies, such as jaw anomalies, scoliosis, lordosis, kyphosis, pre-haemal and caudal vertebrae, within each dietary group (Fig. 7).

Total RNA from larvae samples (average weight per sample 60 mg) was extracted using the Rneasy Mini Kit (Qiagen). Total body tissue was homogenized using the Tissue Lyzer-II (Qiagen, Hilden, Germany) with QIAzol lysis reagent (Qiagen). Samples were centrifuged with chloroform for phase separation (12000 g, 15 min, 4 °C). The upper aqueous phase containing RNA was mixed with 75% ethanol and transferred into an RNeasy spin column where total RNA bonded to a membrane and contaminants were washed away by RW1 and RPE buffers (Qiagen). Purified RNA was eluted with 30 µl of RNase-free water. The quality and quantity of RNA were analyzed using the NanoDrop 1000 Spectrophotometer (Thermo Scientific, Wilmington, DE, USA) and by electrophoresis of total RNA in a 1% agarose gel. Synthesis of cDNA was conducted using the iScript cDNA Synthesis Kit (Bio-Rad) according to manufacturer’s instructions in an iCycler thermal cycler (Bio-Rad, Hercules, CA, USA). Primer efficiency was tested with serial dilutions of a cDNA pool (1, 1:5, 1:10, 1:15, 1:20 and 1:25). Product size of the real-time q PCR amplification was checked by electrophoresis analyses using PB322 cut with HAEIII as a standard. Real-time quantitative PCR was performed in an iQ5 Multicolor Real-Time PCR detection system (Bio-Rad, Hercules, CA, USA) using RAG1 as the house-keeping gene in a final volume of 20 µl per reaction well, and 100 ng of total RNA reverse transcribed to complementary cDNA. Each gene sample was analyzed once per gene. The PCR conditions were the following: 95 °C for 3 min 30 sec followed by 40 cycles of 95 °C for 15 sec, 61 °C for 30 sec, and 72 °C for 30 sec; 95 °C for 1 min, and a final denaturing step from 61 °C to 95 °C for 10 sec. Data obtained were normalized and the Livak method (2−ΔΔCt) used to determine relative mRNA expression levels. Primer design was carried out taking into account the needs of Real time PCR amplification: primer size between 18–22 pb, GC content 50–60%, product size 150–200 pb, avoiding harping or secondary structures, Cs or Gs in 3′ extremity if it was possible and melting temperature of 60 °C ± 2 °C. Primers were designed using Primer3 (v. 0.4.0) software from the conserved regions of each gene obtained by the alignment of gene sequences available on The National Center for Biotechnology Information (NCBI) databases of different fish. Once the primers were designed, the set-up was performed and the correct amplification products were sequenced. Then, the sequences of amplicons were aligned with BLAST NCBI tools searching (Supplementary Fig. S2). Detailed information on primer sequences and accession numbers is presented in Supplementary Table S4.

### Digestive enzyme activities

The head and tail of pikeperch larvae were dissected on a glass maintained on ice (0 °C) to isolate the digestive segment, and the stomach region was separated from the intestinal segments. Pooled samples from each tank were homogenized in 10 volumes (v/w) cold distilled water. Assay of the cytosolic peptidase, leucine alanine peptidase (leu-ala) was performed following the method of Nicholson and Kim^116^ using leucine-alanine (sigma-Aldrich, St LLuis, MO, USA) as substrate. Alkaline phosphatase (AP) and aminopeptidase (N), two enzymes of brush border membrane, were assayed according to Bessey *et al*.^117^ and Maroux *et al*.^118^ using p-nitrophenyl phosphate (Sigma-Aldrich) and L-leucine p-nitroanalide (Sigma-Aldrich) as substrates, respectively. Pepsin was assayed by the method of Worthington^119^ modified by Cuvier-Péres and Kestemont^120^. Trypsin and amylase activities were assayed according to Holm *et al*.^121^ and Metais and Bieth^122^, respectively such as described by Gisbert *et al*.^123^. Protein was determined using the Bradford^124^ procedure. Enzyme activities are expressed as specific activities (U or mU mg protein^−1^).

### Histological analysis

At the beginning and at the end of the experiment 10 pikeperch larvae from each tank were collected and fixed in 10% buffered formalin. For histological process larvae were dehydrated through graded alcohols (70–96°) using a Histokinette 2000 tissue processor (Leica, Nussloch, Germany), then xylene and finally embedded in paraffin wax (Jung Histoembedder, Leica, Nussloch, Germany). Paraffin blocks were sectioned at 5 μm, on a microtome (Leica, RM2135, Leica Instruments, Nussloch, Germany) and stained with hematoxylin, eosin, safran (HES) and examined using light microscopy using a Olympus CX41 binocular microscope (Olympus, Hamburg, Germany), in a range of magnifications (10–40x), connected to an Olympus XC30 camera (Olympus, Hamburg, Germany), which was linked to a computer using image capturing software (CellB®, Olympus, Hamburg, Germany). To assess hepatocyte vacuolization and presence of goblet cells, a three-point scoring system was used (Supplementary Fig. S1), based on a modified method from Betancor *et al*.^125^:

- Score 1: no hepatocyte vacuolization or presence of goblet cells.
- Score 2: mild hepatic vacuolization or presence of goblet cells.
- Score 3: severe hepatocyte vacuolization or presence of goblet cells.

### Biochemical analysis

Representative samples of the experimental diets were analyzed for ash (NMKL, 1991), crude protein 245 (ISO 2005; crude protein; Kjeldahl N × 6·25) and crude lipid^126^. FA analysis of feeds and larvae was done according to previously described^18^. Lipids were extracted by a chloroform/methanol mixture, (2:1 (v/v)^126^ and 40 µl (1 mg mL^−1^) of an internal 23:0 FAME standard from Sigma Aldrich was added. A fixed amount of each feed (5 mg) was weighed and for larval samples (2 × 10 larvae per tank) were weighed and homogenized by a Tissue Tearor probe diameter 4.5 mm, Biospec Products, Inc; Bartlesville, USA. Samples were allowed standing for 24 h in −20 °C followed by thawing and subsequent centrifugation. The supernatant was subsequently transferred to clean GC vials and allowed drying out in a Pierce, reacti-therm heating module at 60 °C, under a continuous flow of nitrogen. Trans esterification of the lipids was done by addition of 1 mL of acetyl chloride in methanol (40:50:10, HPLC quality) at 95 °C. The fatty acid methyl esters were analyzed by gas chromatography–mass spectrometry (GC–MS). Peaks on a given chromatogram were identified by comparison with the retention time of a commercial mix of a known FAME standard, SUPELCO 18919 (4:0–24:0), from SIGMA (St. Louis, MO, USA). Peaks were quantified by means of the target response factor of the fatty acids and 23:0 as internal standard. Fatty acid concentrations were calculated (MSD Chemstation Data Analysis, G1710FA) based on the quantified peaks of the standard series and the samples as well of dry weight of prey and larvae and expressed as ng sample^−1^. A total of 34 fatty acids were analyzed, but only the most relevant are shown (Supplementary Tables S5 and S6).

Ascorbil-2-monophosphate was extracted from feeds using a phosphate buffer and quantitated by reversed-phase HPLC with UV detection as developed by Roche Vitamins Ltd. Vitamin C concentrations were determined at a wavelength of 293 nm and quantification achieved by comparison with tris (cyclohexylammonium) ascorbic acid-2-phosphate (Sigma-Aldrich), used as a reference substance. Vitamin A (Retinoids) on diets was analyzed by HPLC using the method proposed by Takeuchi *et al*.^127^. Vitamins E (α-tocopherol) and D3 were determined using HPLC with UV detection at 293 nm and 254 nm, respectively, as described by McMurray *et al*.^128^ and Takeuchi *et al*.^129^.

Experimental feeds were analyzed for alkali and trace minerals in ICP-MS (iCapQ ICP-MS, Thermo Scientific, Waltham, USA) equipped with an auto sampler (FAST SC-4Q DX, Elemental Scientific, Omaha, USA) according to Julshamn and Brenna^130^.

### Statistics

To determine the best combinations of factors-modalities, each experimental unit was assigned to a global score of interest. This global score was calculated using results of husbandry output variables and was based on the transformation of each output in centered reduced output^58^. Principal component analyses (PCA) were also performed to analyze the global effect of combinations on husbandry output variables.

Main effects and two factor-interactions were then analyzed using Analys software^114^. This method is first based on the detection of potentially active effects using Daniel graphics^131^. It is followed by ANOVA to test these potentially active effects. Significant results (p < 0.05) were finally confirmed by ANOVA (p < 0.05) using Statistica software version 10 (StatSoft Inc., France, 2011), means were compared according to the Tukey post hoc test.

## Supplementary information

Supplementary information
