# Supplementary material for: Key nutritional factors and interactions during larval development of pikeperch (Sander lucioperca)
Source: Sci Rep. 2019 May 8;9:7074. doi: 10.1038/s41598-019-43491-1 (PMC6506547; doi:10.1038/s41598-019-43491-1)
Supplement: Supplementary file 1 — Supplementary information [file 41598_2019_43491_MOESM1_ESM.pdf]

Key nutritional factors and interactions during larval development of pikeperch (*Sander lucioperca*)

Najlae El Kertaoui[1], Ivar Lund[2], Hospice Assogba[1], David Domínguez[3], Maria S. Izquierdo[3], Sebastien Baekelandt[1], Valerie Cornet[1], Syaghalirwa N.M. Mandiki[1], Daniel Montero[3] and Patrick Kestemont[1]

[1]Research Unit in Environmental and Evolutionary Biology (URBE), Institute of Life, Earth & Environment (ILEE), University of Namur, Rue de Bruxelles, 61 - 5000 Namur, Belgium.

[2]Technical University of Denmark, DTU Aqua, Section for Aquaculture, The North Sea Research Centre, P.O. Box 101, DK-9850 Hirtshals, Denmark.

[3]Instituto ECOAQUA, Universidad de Las Palmas de Gran Canaria. Grupo de Investigación en Acuicultura (GIA). Muelle de Taliarte s/n, 35200 Telde, Las Palmas, Canary Islands, Spain.

***Supplementary information**

Table S1: Selected factors and modalities

Factors	Levels	References
**Ca/P**	0.6<br>1.2	- Good performance in pikeperch larvae at Ca/P: 0.5-0.6 even with a lower level of dietary protein compared with other diets [11]<br>- Normal Ca/P for rainbow trout: 0.9-1 [132], but low: 0.5-06 in condition of low Ca availability [133, 134]
**EPA+DHA (%)**	1.25<br>3.5	- Best growth and development in pikeperch larvae fed 1.25% EPA+DHA (1.25% vs 2.64%) [10, 14]<br>- Significant improvement in growth (body weight) in pikeperch larvae fed 2.67 and 3.79% EPA+DHA [19]
**ARA (%)**	0.8<br>1.6	- Best growth and development in pikeperch at 0.8% ARA vs higher level [14]<br>- Good survival and SGR in sea bass: up to 1.2% [27]
**Vitamin E (mg/kg)**	1000<br>3000	- Higher Vit E requirement (3000mg/kg) due to high LC-PUFA needs during larval stages [28, 135]<br>- In seabass larvae: 3000mg/kg adequate for good larval performance, avoid muscular lesions, and improved stress resistance [135, 136]<br>- Requirement measurements depend to interactions of vitamin E with other nutrients [68]
**Vitamin C (mg/kg)**	2000<br>3600	- 3600 mg/kg improved the protection against peroxidation, decreased TBARS contents, spared vitamin E, and reduced the incidence of muscular lesions [30]
**Vitamin A (IU/kg)**	8000<br>30000	- Optimum dietary vitamin A content (based on growth): around 8000–9000 IU VA/kg for Atlantic halibut and Japanese flounder juveniles respectively [137, 138]<br>- Vitamin A toxic level: maximum dose around 45000 IU vitamin A/kg dry diet [139]
**Vitamin D (IU/kg)**	2800<br>28000	- 27600 IU vitamin D3/kg: best result of sea bass larval morphogenesis, and earlier maturation of digestive function [140]<br>- Vitamin D content in commercial diets used in larval rearing: 2500-2800 IU vitamin D3/kg diet, in agreement with the value recommended by NRC [53] (2400 IU VD3/kg).
**Se (mg)**	3<br>12	- Se concentration in fish around 2-3 mg/kg [46]<br>- Up to 11.65 mg Se/kg improved survival rate, stress resistance and promoted the expression of bone formation and mineralization genes in seabream larvae [45]<br>- Se toxicity has been demonstrated at dietary levels of 10-20 mg/kg [141]

Table S2: Aliasing structure considering the 8 dietary variables: Ca/P, EPA+DHA, ARA, vitamin E (vit E), vitamin C (vit C), vitamin D (vitD), vitamin A (vitA), and selenium (Se)

Group	Aliased effects
1	Ca/P*ARA; EPA+DHA*Se; vitE*vitD; vitC*vitA
2	Ca/P*vitE; EPA+DHA*vitC; ARA*vitD; vitA*Se
3	Ca/P*Se; EPA+DHA*ARA; vitE*vitA; vitD*vitC
4	Ca/P*EPA+DHA; ARA*Se; vitE*vitC; vitD*vitA
5	Ca/P*vitD; EPA+DHA*vitA; ARA*vitE; vitC*Se
6	Ca/P*vitA; EPA+DHA*vitD; ARA*vitC; vitE*Se
7	Ca/P*vitC; EPA+DHA*vitE; ARA*vitA; vitD*Se

Table S3: Formulation and the proximate composition (%) of the experimental diets

	D1	D2	D3	D4	D5	D6	D7	D8	D9	D10	D11	D12	D13	D14	D15	D16
**Ingredients (%)**																
MicroNorse	25.0	25.0	25.0	25.0	25.0	25.0	25.0	25.0	25.0	25.0	25.0	25.0	25.0	25.0	25.0	25.0
CPSP 90	8.00	8.00	8.00	8.00	8.00	8.00	8.00	8.00	8.00	8.00	8.00	8.00	8.00	8.00	8.00	8.00
Squid meal	17.0	17.0	17.0	17.0	17.0	17.0	17.0	17.0	17.0	17.0	17.0	17.0	17.0	17.0	17.0	17.0
Krill meal (Aker Biomarine)	8.00	8.00	8.00	8.00	8.00	8.00	8.00	8.00	8.00	8.00	8.00	8.00	8.00	8.00	8.00	8.00
Fish gelatin	2.00	2.00	2.00	2.00	2.00	2.00	2.00	2.00	2.00	2.00	2.00	2.00	2.00	2.00	2.00	2.00
Wheat Gluten	5.00	5.00	5.00	5.00	5.00	5.00	5.00	5.00	5.00	5.00	5.00	5.00	5.00	5.00	5.00	5.00
Potato starch gelatinised (Pregeflo)	10.45	12.95	9.15	13.37	8.95	13.27	9.49	13.85	8.68	13.04	9.14	13.46	9.04	13.26	9.58	12.08
Fish oil - SAVINOR	1.20	1.20	0.80	0.80	1.15	1.15	0.00	0.00	1.20	1.20	0.80	0.80	1.15	1.15	0.00	0.00
Incromega DHA 500TG	0.00	0.00	3.40	3.40	0.00	0.00	3.58	3.58	0.00	0.00	3.40	3.40	0.00	0.00	3.58	3.58
VEVODAR	2.10	2.10	2.10	2.10	4.25	4.25	4.25	4.25	2.10	2.10	2.10	2.10	4.25	4.25	4.25	4.25
Soybean oil	3.00	3.00	0.00	0.00	1.05	1.05	0.00	0.00	3.00	3.00	0.00	0.00	1.05	1.05	0.00	0.00
Vit & Min Premix PV02	0.20	0.20	0.20	0.20	0.20	0.20	0.20	0.20	0.20	0.20	0.20	0.20	0.20	0.20	0.20	0.20
Lutavit C35	0.58	1.02	1.02	0.58	0.58	1.02	1.02	0.58	1.02	0.58	0.58	1.02	1.02	0.58	0.58	1.02
Lutavit E50	0.20	0.20	0.20	0.20	0.20	0.20	0.20	0.20	0.60	0.60	0.60	0.60	0.60	0.60	0.60	0.60
Rovimix A (5000000 IU/kg)	0.00	0.00	0.44	0.44	0.44	0.44	0.00	0.00	0.44	0.44	0.00	0.00	0.00	0.00	0.44	0.44
Rovimix D3 (5000000 IU/kg)	0.015	0.51	0.015	0.51	0.51	0.015	0.51	0.015	0.51	0.015	0.51	0.015	0.015	0.51	0.015	0.51
Brewer's yeast	5.00	5.00	5.00	5.00	5.00	5.00	5.00	5.00	5.00	5.00	5.00	5.00	5.00	5.00	5.00	5.00
Choline chloride	0.50	0.50	0.50	0.50	0.50	0.50	0.50	0.50	0.50	0.50	0.50	0.50	0.50	0.50	0.50	0.50
Soy lecithin - Powder	6.00	6.00	6.00	6.00	6.00	6.00	4.50	4.50	6.00	6.00	6.00	6.00	6.00	6.00	4.50	4.50
Binder (sodium alginate)	1.00	1.00	1.00	1.00	1.00	1.00	1.00	1.00	1.00	1.00	1.00	1.00	1.00	1.00	1.00	1.00
NaH2PO4	4.20	0.35	4.20	0.35	4.20	0.35	4.20	0.35	4.20	0.35	4.20	0.35	4.20	0.35	4.20	0.35
SelPlex - Se yeast	0.05	0.47	0.47	0.05	0.47	0.05	0.05	0.47	0.05	0.47	0.47	0.05	0.47	0.05	0.05	0.47
L-Taurine	0.50	0.50	0.50	0.50	0.50	0.50	0.50	0.50	0.50	0.50	0.50	0.50	0.50	0.50	0.50	0.50
**Proximate composition(%)**																
Moisture*	6.40	6.20	6.20	6.30	6.50	6.30	6.30	6.50	6.40	6.40	6.30	6.50	6.60	6.40	6.30	6.50
Crude protein*	51.1	51.3	51.5	51.1	51.3	51.1	51.1	51.2	51.0	51.3	51.3	51.0	51.2	51.1	51.1	51.2
Crude fat*	17.4	17.5	17.5	17.6	17.6	17.7	17.7	17.5	17.5	17.5	17.6	17.5	17.6	17.6	17.6	17.5
Crude ash*	7.60	6.60	7.6	6.6	7.50	6.70	7.60	6.60	7.50	6.50	7.60	6.60	7.60	6.60	7.60	6.60

Phosphorus*	1.97	1.06	1.99	1.07	1.96	1.07	2.01	1.05	1.98	1.06	2.01	1.07	1.95	1.06	2.03	1.05
Calcium*	1.24	1.26	1.25	1.24	1.25	1.24	1.25	1.24	1.24	1.24	1.26	1.23	1.23	1.24	1.24	1.24
Selenium*	4.00	14.00	14.00	4.00	15.00	4.00	4.00	14.00	5.00	14.00	14.00	4.00	14.0	4.00	4.00	14.00
Vitamin A**	7794	7775	28933	28896	28866	28917	7825	7794	28877	28891	7816	7777	7800	7814	28891	28843
Vitamin C†	1944	3428	3415	1952	1941	3409	3418	1948	3417	1933	1947	3416	3401	1946	1956	3417
Vitamin D3**	2893	28544	2835	28707	28006	2794	28430	2829	28397	2881	28208	2884	2831	28777	2848	28805
Vitamin E†	997	1006	992	1004	997	1001	1006	994	2952	2947	2956	2946	2938	2871	2967	2897

*dietary content per g/100g; **vitamin A&D presented per IU/kg †vitamin C&E presented per mg/kg

Table S4: Sequences of primers used for gene expression analysis

Gene	Nucleotide Sequence	Accesion nº	Amplicon	Tm
Rag1	F: 5’-AGCCAAAGCCAAACTCAGAA-3’	KC819903	150	60
	R: 5’-TCACGCACCATCTTCTCATC-3’			
Twist2	F: 5’-CCCCTGTGGATAGTCTGGTG-3’	--	226	60
	R: 5’-GACTGAGTCCGTTGCCTCTC-3’			
Mef2c	F: 5’-GCGAAAGTTTGGCCTGATGA-3’	--	180	60
	R: 5’-TCAGAGTTGGTCCTGCTCTC-3’			

Table S5: Fatty acid composition (% of TFA) of the 16 experimental feed types

	D1	D2	D3	D4	D5	D6	D7	D8	D9	D10	D11	D12	D13	D14	D15	D16
16:0	13.4	13.2	12.2	12.80	13.30	13.10	10.40	10.70	13.50	13.50	12.00	12.20	13.30	13.50	10.60	10.30
18:0	4.10	4.10	3.50	3.60	4.20	4.20	3.90	3.90	4.10	4.10	3.50	3.40	4.20	4.30	3.90	4.00
Total SFA	27.0	27.10	25.80	26.6	30.10	29.9	26.0	26.50	27.30	27.10	26.10	25.90	30.00	30.60	26.20	26.10
16:1 (n-7)	2.60	2.70	2.70	2.70	2.70	2.60	2.10	2.20	2.60	2.60	2.60	2.60	2.60	2.70	2.10	2.10
18:1 (n-9)	15.50	15.50	11.80	11.90	12.90	12.70	10.10	10.30	15.40	15.40	11.50	11.50	12.90	12.90	10.10	9.90
Total MUFAs	24.35	23.65	21.85	21.40	21.40	21.10	18.30	18.60	23.70	23.90	21.00	21.50	21.00	21.20	18.10	18.00
18:2 (n-6)	16.50	15.70	12.80	13.5	14.30	14.10	10.60	10.60	16.50	16.40	12.50	12.10	14.60	14.30	11.00	10.30
20:4 (n-6) ARA	9.00	9.40	10.30	10.0	17.90	18.20	17.80	17.70	9.20	9.10	11.30	10.30	17.70	18.00	17.40	18.10
Total (n-6) PUFA	26.40	27.00	25.20	26.00	35.40	35.50	31.90	31.50	27.50	27.50	26.20	24.60	35.40	35.50	31.90	31.70
18:3 (n-3)	14.00	14.00	1.40	1.40	5.80	5.60	1.10	1.10	13.70	14.0	1.40	1.30	5.70	5.80	1.10	1.00
20:3 (n-3)	0.80	0.10	0.20	0.30	0.10	0.20	0.30	0.30	0.20	0.20	0.30	0.30	0.20	0.20	0.20	0.20
20:5 (n-3) EPA	3.90	3.90	6.50	6.30	3.90	3.90	5.40	5.40	3.90	3.90	6.20	6.60	3.90	3.50	5.30	5.30
22:6 (n-3) DHA	4.80	4.90	19.80	18.80	4.80	5.00	17.70	17.30	4.90	4.70	19.20	19.90	4.80	4.60	17.40	17.90
Total (n-3) HUFA	9.85	9.25	27.55	26.45	9.15	9.45	24.25	23.85	9.35	9.15	26.75	28.05	9.25	8.65	23.75	24.35
DHA/EPA	1.23	1.26	3.05	2.98	1.23	1.28	3.28	3.20	1.26	1.21	3.10	3.02	1.23	1.31	3.28	3.38
DHA/ARA	0.53	0.52	1.92	1.88	0.27	0.27	0.99	0.98	0.53	0.52	1.70	1.93	0.27	0.26	1.00	0.99
EPA/ARA	0.43	0.41	0.63	0.63	0.22	0.21	0.30	0.31	0.42	0.43	0.55	0.64	0.22	0.19	0.30	0.29
Oleic/DHA	3.23	3.16	0.60	0.63	2.69	2.54	0.57	0.60	3.14	3.28	0.60	0.58	2.69	2.80	0.58	0.55
(n-3)/(n-6)	0.93	0.91	1.19	1.11	0.44	0.44	0.82	0.81	0.87	0.87	1.11	1.23	0.44	0.43	0.80	0.82

Table S6: Larval fatty acids composition (% of TFA) of pikeperch larvae fed different experimental diets (a pool of 10 larvae per treatment)

	**D1**	**D2**	**D3**	**D4**	**D5**	**D6**	**D7**	**D8**	**D9**	**D10**	**D11**	**D12**	**D13**	**D14**	**D15**	**D16**
Total SFA	26.66	28.42	23.23	23.93	27.53	25.9	26.31	22.39	28.53	27.65	24.69	24.51	27.29	28.07	23.04	24.51
Total (n-9)	6.33	6.56	4.46	4.52	4.93	4.84	4.62	3.81	6.40	6.04	4.71	4.63	5.24	5.77	4.08	4.41
Total MUFAs	6.42	6.60	4.53	4.55	4.28	4.91	4.73	3.84	6.47	6.1	4.78	4.69	5.3	5.83	4.14	4.45
18:2 (n-6)	15.27	15.33	10.94	10.32	10.82	11.26	10.22	8.34	14.52	13.42	11.28	10.92	12.26	14.97	9.50	9.61
18:3 (n-6)	0.50	0.55	0.39	0.36	0.63	0.64	0.66	0.52	0.55	0.46	0.42	0.38	0.71	0.80	0.61	0.62
20:4 (n-6) ARA	12.01	12.61	10.99	11.06	21.41	20.48	19.97	17.39	13.73	13.62	12.32	11.06	21.01	20.85	19.17	20.14
Total (n-6)	28.36	29.12	22.78	22.74	33.69	33.22	31.77	26.94	29.60	28.16	24.53	23.41	35.03	37.68	30.09	31.28
Total (n-3)	36.02	33.11	46.26	45.19	31.27	33.42	34.23	44.50	32.92	35.04	42.42	44.47	29.86	26.01	29.98	37.45
18:3 (n-3)	6.43	5.79	0.78	0.82	1.97	2.09	0.71	0.72	5.23	5.26	0.83	1.23	2.39	2.95	0.68	0.71
20:5 (n-3) EPA	5.03	5.12	5.52	5.74	4.61	4.46	5.42	4.76	5.19	5.31	6.16	5.88	4.6	4.69	5.27	5.31
22:6 (n-3) DHA	24.35	21.94	39.57	38.28	24.28	26.58	27.65	38.39	22.08	24.12	35.19	37.08	22.45	17.83	33.71	30.92
Total (n-3) LC-PUFA	29.59	27.32	45.48	44.37	39.3	31.33	33.52	43.78	27.69	29.78	41.59	43.24	27.47	23.06	39.30	36.74
DHA/EPA	4.84	4.29	7.17	6.67	5.27	5.96	5.10	8.07	4.25	4.54	5.71	6.31	4.88	3.80	6.40	5.82
EPA/ARA	0.42	0.41	0.50	0.49	0.22	0.22	0.27	0.27	0.38	0.39	0.50	0.51	0.22	0.22	0.27	0.26

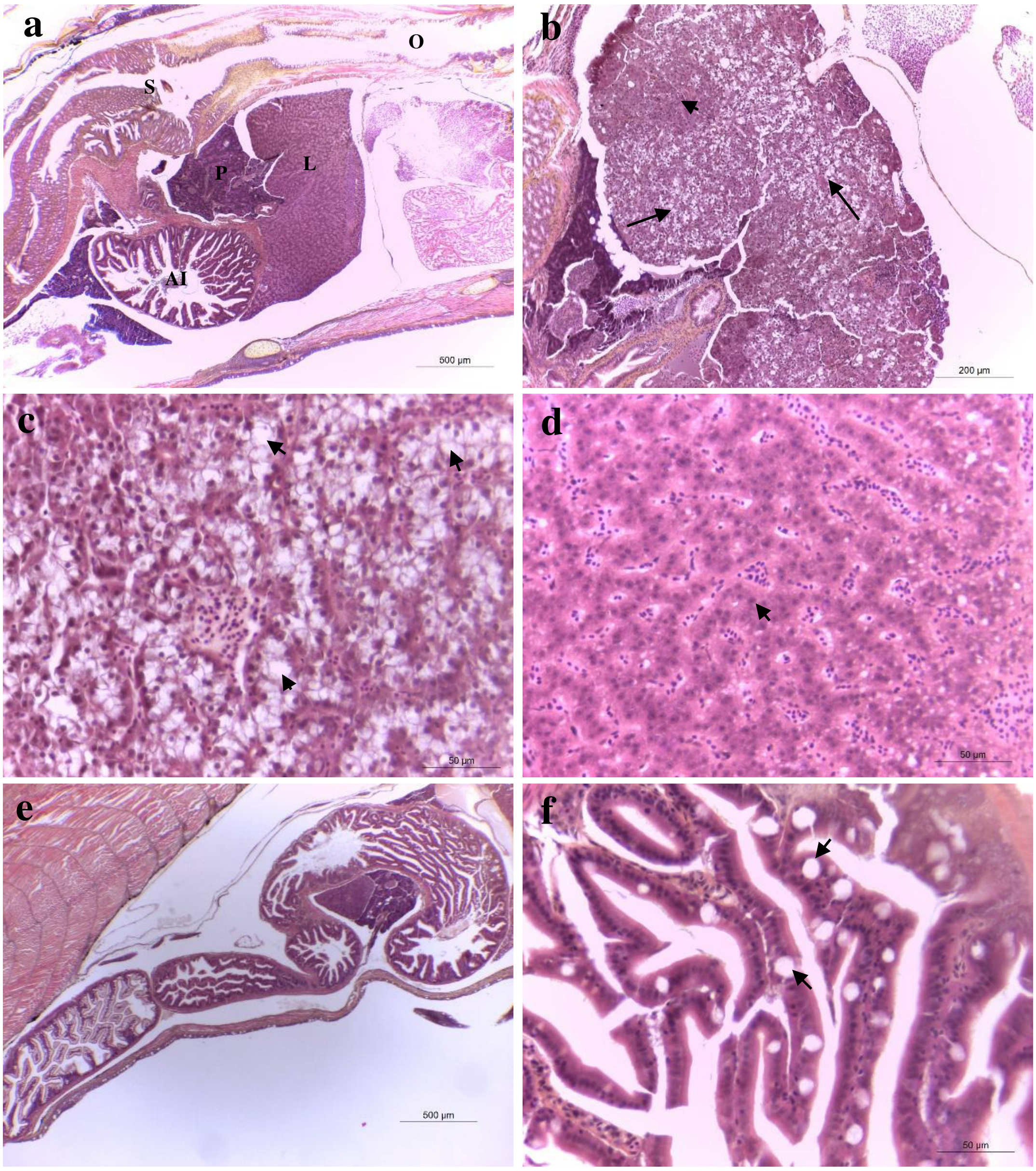

Figure S1. Sections of larvae intestine and liver of pikeperch *Sander lucioperca* from different treatments (10 larvae evaluated per treatment). HES stain. (a) longitudinal section of pikeperch larvae digestive tract: (AI) anterior intestine, (O) oesophagus, (L) liver, (P) pancreas, (S) stomach. (b) liver larvae section showing different degrees of hepatocyte vacuolization levels: very scarce hepatocyte vacuolization area (small arrow) and high lipid vacuoles deposition area (large arrows). (c) high magnification section showing cytoplasmic vacuolization of hepatocytes (arrows). (d) histological structure of hepatic lobule showing condensed hepatocytes with centred nucleus (arrow) and marked cytoplasm staining. (e) whole intestine of 39 dph pikeperch larvae. (f) abundance of goblet cells in the intestine sections (arrows).

a.

```
Sl-mef2c                      GCGAAAGTTTGGCCTGATGAAGAAGGCGTATGAGCTGCAGTGTGCTGTGTGACTGTGAGA      60
XR_003213332.1-On-mef2cX1     GAGAAAGTTTGGCCTGATGAAGAAGGCGTATGAGCT-GAGCGTGCTGTGTGACTGTGAGA      59
XR_003213335.1-Onmef2cX4      GAGAAAGTTTGGCCTGATGAAGAAGGCGTATGAGCT-GAGCGTGCTGTGTGACTGTGAGA      59
XM_010782446.1-Nc-mef2cX5     GCGAAAGTTTGGCCTGATGAAGAAGGCGTATGAGCT-GAGTGTGCTGTGTGACTGTGAGA      59
XM_010782445.1-Nc-mef2cX4     GCGAAAGTTTGGCCTGATGAAGAAGGCGTATGAGCT-GAGTGTGCTGTGTGACTGTGAGA      59
XM_028578836.1-Pf-mef2cX7     GCGAAAGTTTGGCCTGATGAAGAAGGCGTATGAGCT-GAGTGTGCTGTGTGACTGTGAGA      59
XM_028578833.1-Pf-mef2cX4     GCGAAAGTTTGGCCTGATGAAGAAGGCGTATGAGCT-GAGTGTGCTGTGTGACTGTGAGA      59
                              * **********************************  ** *******************

Sl-mef2c                      TTGCCCTGATCATCTTCAATAGCACCAACAAGCTGTTCCAGTATGCCAGCACAGACATGG      120
XR_003213332.1-On-mef2cX1     TTGCCCTGATCATCTTCAACAGCACCAACAAGCTGTTCCAGTATGCCAGCACAGACATGG      119
XR_003213335.1-Onmef2cX4      TTGCCCTGATCATCTTCAACAGCACCAACAAGCTGTTCCAGTATGCCAGCACAGACATGG      119
XM_010782446.1-Nc-mef2cX5     TTGCCCTGATCATCTTCAATAGCACCAACAAGCTGTTCCAGTATGCCAGCACAGACATGG      119
XM_010782445.1-Nc-mef2cX4     TTGCCCTGATCATCTTCAATAGCACCAACAAGCTGTTCCAGTATGCCAGCACAGACATGG      119
XM_028578836.1-Pf-mef2cX7     TTGCCCTGATCATCTTCAATAGCACCAACAAGCTGTTCCAGTATGCCAGCACAGACATGG      119
XM_028578833.1-Pf-mef2cX4     TTGCCCTGATCATCTTCAATAGCACCAACAAGCTGTTCCAGTATGCCAGCACAGACATGG      119
                              ****************** *****************************************

Sl-mef2c                      ACAAGGTCCTGCTTAAATACACCGAGTACAACGAGCCCCATGAGAGCAGGACCAACTCTG      180
XR_003213332.1-On-mef2cX1     ACAAGGTCCTGCTTAAATACACCGAGTACAATGAGCCCCATGAGAGCAGGACCAACTCTG      179
XR_003213335.1-Onmef2cX4      ACAAGGTCCTGCTTAAATACACCGAGTACAATGAGCCCCATGAGAGCAGGACCAACTCTG      179
XM_010782446.1-Nc-mef2cX5     ACAAGGTCCTGCTTAAATACACCGAGTACAACGAGCCCCATGAGAGCAGGACCAACTCAC      179
XM_010782445.1-Nc-mef2cX4     ACAAGGTCCTGCTTAAATACACCGAGTACAACGAGCCCCATGAGAGCAGGACCAACTCAC      179
XM_028578836.1-Pf-mef2cX7     ACAAGGTCCTGCTTAAATACACAGAGTACAATGAGCCCCATGAGAGCAGGACCAACTCTG      179
XM_028578833.1-Pf-mef2cX4     ACAAGGTCCTGCTTAAATACACAGAGTACAATGAGCCCCATGAGAGCAGGACCAACTCTG      179
                              ********************** ******** **************************

Sl-mef2c                      A 181
XR_003213332.1-On-mef2cX1     A 180
XR_003213335.1-Onmef2cX4      A 180
XM_010782446.1-Nc-mef2cX5     C 180
XM_010782445.1-Nc-mef2cX4     C 180
XM_028578836.1-Pf-mef2cX7     A 179
XM_028578833.1-Pf-mef2cX4     A 180
```

b.

```
Sl-Twist2                     CCCCTGTGGATAGTCTGGTGACCAGCGAGGAGGAGCTGGACAGACAGCAGAAACGCTTCG      60
XM_028591816.1-Pf-Twist2      CCCCAGTGGATAGTCTGGTGACCAGCGAGGAGGAGCTGGACAGAAGGCAGAAACGCTTCG      60
XM_023292754.1-Ao-twist2      CCCCTGTGGATAGTCTGGTGACCAGCGAGGAGGAGCTGGACAGGCAGCAGAAGCGCTTCG      60
XM_005450442.4-On-Twist2      CCCCTGTGGATAGTCTGGTGACCAGCGAGGAGGAGCTGGACAGACAGCAGAAAAGGTTCG      60
                              **** **************************************   ******  * ****

Sl-Twist2                     CGGGGAAGAGGAGACAAAGCAAAAAGTCCAGCGAGGACAGCAGCGGCGGCAGCAGCCCGG      120
XM_028591816.1-Pf-Twist2      CGGGGAAGAGGAGACAAAGCAAAAAGTCCAGCGAGGACAGCAGCGGCGGCAGCAGCCCGG      120
XM_023292754.1-Ao-twist2      CGAGGAAGAGGAGACACAGTAAAAAGTCCAGCGAGGACAGCAGCG---GCAGCAGCCCGG      117
XM_005450442.4-On-Twist2      CGAGGAAGAGGAGGCACAGCAAAAAGTCCAGCGACGACAGCAGCG---GGAGCAGCCCGG      117
                              ** ********** ** ** ************** **********   * **********

Sl-Twist2                     GTCCGGTTAAACGGGTTAAAAAGGCGAGTCCGAGCAGCAATCAGTCGTACGAGGAGCTGC      180
XM_028591816.1-Pf-Twist2      GTCCGGTTAAACGGGTTAAAAAGGCGAGTCCGAGCAGCAATCAGTCGTACGAGGAGCTGC      180
XM_023292754.1-Ao-twist2      GGCCGGTGAAGCGGGGCAAGAAGCCGAGTCCGAGCAGCACTCAGTCGTACGAGGAGCTGC      177
XM_005450442.4-On-Twist2      GTCCGGTGAAACGGGGGAAGAAGCCGAGTCCGAGCAGCACTCAGTCGTACGAGGAGCTGC      177
                              * ***** ** ****  ** *** *************** ********************

Sl-Twist2                     AGAACCAGCGGGTCCTGGCCAACGTCCGGGAGAGGCAACGGACTCAGTC         229
XM_028591816.1-Pf-Twist2      AGAACCAGCGGGTCCTGGCCAACGTCCGGGAGAGGCAACGGACTCAGTC         229
XM_023292754.1-Ao-twist2      AGAACCAGCGGGTCCTGGCCAACGTCCGGGAGAGGCAACGGACTCAGTC         226
XM_005450442.4-On-Twist2      AGAACCAGCGGGTCCTGGCCAACGTCCGGGAGAGGCAACGGACTCAGTC         226
                              *************************************************
```

Figure S2. (a) Sequence alignment of myocyte enhancer factor 2C (mef2c) genes from *Sander* lucioperca, *Oreochromis niloticus* mef2c variant X4 (XR_003213332.1) and X5 (XR_003213335.1), *Notothenia coriiceps* variant X5 (XM_010782446.1) and X5 XM_010782445.1, *Perca flavescens* variant X7 (XM_028578836.1) and X5 (XM_028578833.1). (b) Sequence alignment of twist-related protein 2-like genes from with *Sander luciopera*, *Perca flavescens* (XM_028591816.1), *Amphiprion ocellaris* (XM_023292754.1), *Oreochromis nolíticus* (XM_005450442.4). Primer sequences used for qPCR amplification are underlined in the gene sequences of *S. lucioperca*,* represents the similarity of sequences.

References

132. Rodehutscord, M. Response of rainbow trout (*Oncorhynchus mykiss*) growing from 50 to 200 g to supplements of dibasic sodium phosphate in a semipurified diet. *J Nutr.* **126**, 324–331 (1996).

133. Vielma, J., Lall, S., Koskela, J., & Mattila, P. Influence of low dietary cholecalciferol intake on phosphorus and trace element metabolism by rainbow trout (*Oncorhynchus mykiss*, Wal- baum). *Comp Biochem Physiol A: Molecular & Integrative Physiology.* **122**, 117–125 (1999).

134. Antony Jesu Prabhu, P. et al. Post-prandial changes in plasma mineral levels in rainbow trout fed a complete plant ingredient based diet and the effect of supplemental di-calcium phosphate. *Aquaculture.* **430**, 34-43 (2014).

135. Atalah, E. et al. Enhancement of gilthead seabream (*Sparus aurata*) larval growth by dietary vitamin E in relation to two different levels of essential fatty acids. *Aquac Res.* **43**, 1816-1827 (2012).

136. Betancor, M.B. et al. α-tocopherol in weaning diets for European sea bass, *Dicentrarchus labrax* L. improves survival and reduces tissue damage caused by excess dietary DHA contents. *Aquac Nutr.* **17(2)**, 112-122 (2011).

137. Moren, M. et al. An optimum level of vitamin A supplements for Atlantic halibut (*Hipoglossus hipoglossus* L.) juveniles. *Aquaculture.* **235**, 587–599 (2004).

138. Hernández, L.H.H. et al. Dietary vitamin A requirements of juvenile Japanese flounder *Paralichthys olivaceus*. *Aquac Nutr.* **11**, 3–9 (2005).

139. Cahu, C., Zambonino Infante, J. & Takeuchi, T. Nutritional components affecting skeletal development in fish larvae. *Aquaculture.* **227**, 245–258 (2003).

140. Darias, M.J. et al. Dietary vitamin D3 affects digestive system ontogenesis and ossification in European sea bass (*Dicentrachus labrax*, Linnaeus, 1758). *Aquaculture*. **298**, 300–307 (2010).

141. NRC Nutrient Requirements of Fish. National Research Council, The National Academy Press, Washington, DC (2011).
